# LLM-based feature selection and counterfactual explanations applied to functional connectivity analysis in schizophrenia

**DOI:** 10.3389/fnins.2025.1732013

**Published:** 2026-01-12

**Authors:** Xinyan Yuan, Tiantian Chen, Yanyan He, Lingling Gu, Ying Sun, Shaolong Wei

**Affiliations:** 1 School of Artificial Intelligence, Jiangsu Vocational College of Business, Nantong, China; 2 School of Artificial Intelligence and Computer Science, Nantong University, Nantong, China

**Keywords:** counterfactual explanation, feature selection, functional connectivity, large language model, schizophrenia

## Abstract

**Introduction:**

Schizophrenia (SZ) is a complex psychiatric disorder whose neural mechanisms are still unclear. Functional connectivity (FC) provides a unique perspective for understanding its pathology, but its high-dimensional nature poses significant challenges for feature selection and model interpretation. Traditional feature selection methods, while predictive, lack the integration of prior neuroscience knowledge, resulting in limited clinical relevance.

**Methods:**

To address this, we propose an innovative framework that combines feature selection guided by a large language model (LLM) with counterfactual explanation. This framework leverages brain disease knowledge encoded by the LLM to guide dimensionality reduction of high-dimensional FC, ensuring that selected features are both statistically significant and biologically plausible. Counterfactual explanations are then used to generate causal intervention examples, which are then translated by the LLM into intuitive explanations in natural language, providing understandable and actionable clinical insights for individual patients or physicians.

**Results:**

We validate our approach on five real-world SZ datasets and demonstrate that it not only improves model classification performance but also provides new insights into SZ analysis.

**Discussion:**

The LLM-based FC analysis method proposed in this study demonstrates good feature selection and interpretability on multiple SZ datasets. Its main advantage is its ability to effectively screen key FC features for brain regions. However, this method has some limitations, such as being difficult to directly apply clinically due to data heterogeneity, being unable to accurately locate individual FC abnormalities, and the hyperparameters for counterfactual generation not yet being optimized.

## 1 Introduction

Schizophrenia (SZ) is a severe mental illness characterized by clinical manifestations including hallucinations, delusions, and disorganized thinking, leading to significant impairment in patients' social functioning (McCutcheon et al., 2020, 2023). Despite its widespread global impact, the underlying neuropathological mechanisms remain largely unknown, posing significant challenges for objective diagnosis and effective intervention (Insel, 2010; Fišar, 2023). Current research suggests that abnormal connectivity between functional brain regions is a key characteristic of SZ (Zhang et al., 2021). Functional connectivity (FC), which reflects the coordinated activity between different brain regions, provides a unique perspective for understanding the pathological mechanisms of SZ (Chyzhyk et al., 2015; Zhu et al., 2024). With the advancement of neuroimaging technology, functional magnetic resonance imaging (fMRI) has become a standard method for studying brain activity and connectivity patterns, making FC an effective tool for investigating SZ (Shen et al., 2010; Lynall et al., 2010).

However, analyzing FC data presents significant methodological challenges. FC is typically represented as a high-dimensional matrix whose number of elements depends on the number of brain regions mapped by the brain atlas (Li et al., 2021; Mhiri and Rekik, 2020). This results in feature dimensions reaching thousands or even tens of thousands for each subject (Ting et al., 2018). First, redundant information in the data can lead to model overfitting, resulting in poor generalization of diagnosis and prediction (Tian and Zalesky, 2021). Second, high-dimensional data makes it difficult for traditional statistical methods to effectively extract important features related to SZ (Naheed et al., 2020). Therefore, reducing dimensionality while maintaining data validity and information content has become a key issue in current FC data analysis.

Although traditional feature selection methods (such as LASSO, MRMR, and XGBoost) have demonstrated certain predictive capabilities in high-dimensional data (Wang et al., 2022), they generally rely on purely data-driven statistical indicators and lack explicit modeling of the biomedical knowledge behind the features, resulting in the selected features being difficult to interpret or disconnected from clinical intervention (Hua et al., 2009; des Touches et al., 2023). In recent years, with breakthroughs in natural language understanding and knowledge reasoning using large language models (LLMs), researchers have begun exploring the use of LLMs as a source of prior knowledge in feature selection (Bal-Ghaoui and Sabri, 2025; Mutian et al., 2025; Choi et al., 2022). By incorporating the rich knowledge of brain diseases from LLMs, the feature selection process becomes more relevant to real-world medical contexts, enabling the identification of features that are highly relevant to clinical diagnosis (Oh and Lee, 2025). These features are also easier to interpret and integrate with clinical interventions.

**Figure 1:**
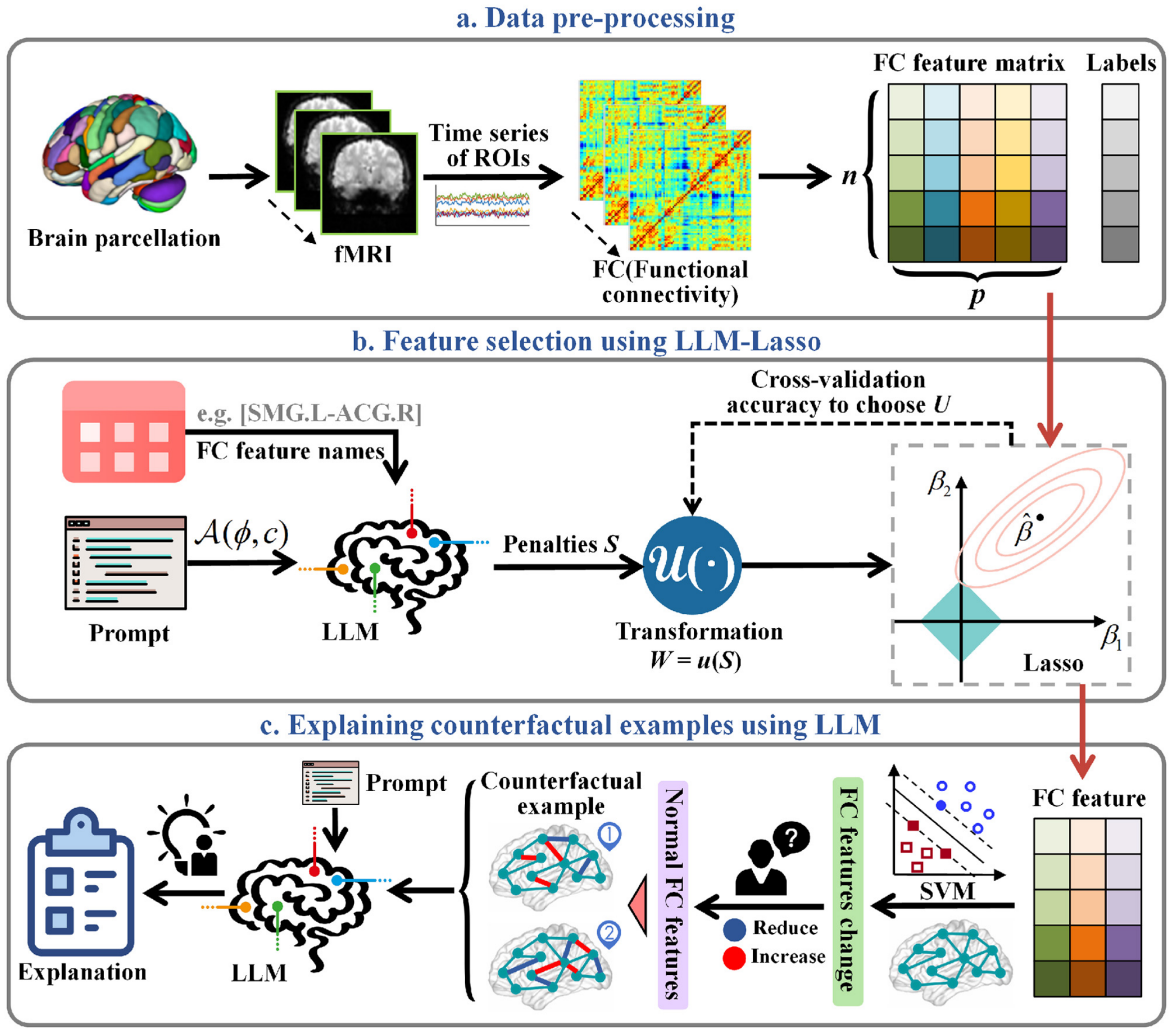
Illustration of our proposed schizophrenia analysis method, including **(a)** data pre-processing, **(b)** Feature selection using LLM-Lasso, **(c)** Explaining counterfactual examples using LLM.

To address these challenges, this study proposes an innovative framework that combines LLM-guided feature selection with counterfactual explanation techniques, achieving a closed loop between knowledge-guided feature selection and causal-guided interpretation in FC analysis of SZ. A schematic diagram of our proposed method is shown in Figure 1. Specifically, we first preprocess the rs-fMRI data and extract the upper triangular elements to construct FC feature matrix. Next, we develop a robust feature selection framework based on the LLM. By transforming the LLM's embedded prior knowledge of brain disease into connection-specific penalty weights, we effectively narrow the search space and prioritize FC features with potential intervention value. Finally, we employ the counterfactual explanation model to generate multiple sets of counterfactual examples for schizophrenia patients. Specifically, we fine-tune the patients' abnormal FC features to healthy individuals. These examples are then fed into the LLM to generate intuitive and easily understandable final explanations. We validate our method on five real-world SZ datasets, demonstrating that it not only improves model interpretability but also provides new insights into SZ analysis.

## 2 Related work

Counterfactual explanations, as an explainable artificial intelligence method, have garnered widespread attention in academia and industry in recent years (Wang et al., 2021). Their core idea is to generate a hypothetical input sample that alters the model's prediction, thereby revealing the key rationale behind the model's decision (Verma et al., 2024). Unlike interpretable methods based on feature importance or model structure, counterfactual explanations are closer to human intuition and can present causal explanations to end users in the form of if-then explanations.

Wachter et al. (2017) were the first to systematically propose counterfactual explanations. They framed the problem as an optimization problem: achieving the desired change in model output with the smallest possible input perturbation. This approach emphasizes the proximity, effectiveness, and comprehensibility of explanations, laying the foundation for subsequent research. Subsequently, researchers have expanded on counterfactual explanations from various perspectives. For example, Mothilal et al. (2020) proposed a method for generating multiple and diverse counterfactual explanations to avoid the potential bias of a single explanation. Ustun et al. (2019) focused on feasibility when generating counterfactuals, ensuring that the proposed input changes are actually feasible for users in the real world. Furthermore, Poyiadzi et al. (2020) introduced causal constraints to enhance the causal plausibility of counterfactual explanations.

In recent years, counterfactual explanations have been widely applied in various fields, including financial risk assessment (Cheng et al., 2020), medical diagnosis (Richens et al., 2020), image classification (Khorram and Fuxin, 2022), and recommender systems (Kaffes et al., 2021). In the image domain, researchers use generative models (such as GANs) to generate visually plausible counterfactual images (Melistas et al., 2024). In the text domain, researchers achieve explanations by perturbing keywords or sentence structure (Yang et al., 2020). In this paper, we can make a counterfactual claim that *if the abnormal FC between brain regions in patients with SZ is adjusted to normal ranges, their condition may be closer to that of healthy individuals*. This kind of decision-making is very useful in medicine, helping doctors evaluate the potential effects of different treatment options, especially for brain diseases.

## 3 Materials and methods

### 3.1 Schizophrenia dataset

This study uses five public schizophrenia datasets containing 773 subjects, including the Center for Biomedical Research (COBRE) dataset, Huaxi dataset, Nottingham dataset, Taiwan dataset, and Xiangya dataset. These subjects have the following requirements: (i) no other Diagnostic and Statistical Manual of Mental Disorders (DSMIV) disease exists; (ii) no history of drug abuse; (iii) no clinically significant head trauma. Table 1 summarizes the demographic and clinical characteristics of subjects of these datasets.

**Table 1:** Demographic and clinical information of subjects in five datasets.

**Datasets**	**Class**	**Gender(M/F)**	**P-value of gender**	**Age (years)**	***P*-value of age**
COBRE	NC	46/21	0.1927	34.82 + 11.28	0.3987
	SZ	42/11		36.75 + 13.68	
Huaxi	NC	79/71	0.6748	27.80+12.50	1.000
	SZ	80/81		27.80 + 12.50	
Nottingham	NC	26/10	0.2277	33.38 + 8.98	0.9855
	SZ	27/5		33.34 + 9.05	
Taiwan	NC	25/37	0.2329	29.87 + 8.62	0.2847
	SZ	35/34		31.59 + 9.60	
Xiangya	NC	35/25	0.9333	27.17 + 6.64	0.1025
	SZ	49/34		23.37 + 7.83	

NC, Normal control; SZ, Schizophrenia.

### 3.2 Data pre-processing

Rs-fMRI data are collected using three different types of scanners: 3-T Siemens Tim-Trio scanner with an eight- or 12-channel head coil (COBRE, Taiwan and Xiangya), 3-T General Electric MRI scanner (Huaxi), and 3-T Philips Achieva MRI scanner (Nottingham). The rs-fMRI data are preprocessed using SPM 8 and the Data Processing Assistant for Resting-State fMRI (DPARSF) according to standard procedures. The following steps are performed: (i) removing the first 10 volumes, (ii) slice timing correction, (iii) head motion correction, (iv) regress out the nuisance covariates, (v) normalized to standardized space, (vi) voxel-wise bandpass filtering, (vii) normalization of anatomical images to MNI template space, and (viii) smoothing with a 4 mm Full Width at Half Maximum (FWHM) Gaussian kernel. After processing, the nodes of the brain network are defined according to the Anatomical Automatic Labeling (AAL) template, and the pairwise similarities between the node time series are calculated as the connecting edges of the brain network.

Next, let $A_{i}^{F}\in {\mathbb{R}}^{N\times N}$ be the connectivity matrix of the functional brain network, *N* be the number of regions of the brain network, *i* = 1, 2, ..., *n*, and *n* be the number of subjects. We take the upper triangular elements of the $A_{i}^{F}$ matrix as features and represent them as vectors $x_{i}\in {\mathbb{R}}^{1\times p}$, $p=\frac{N\left(N-1\right)}{2}$, and *y*_*i*_ is the label of the *i*-th subject. Therefore, the FC feature matrix of all subjects can be represented as $X={\left[x_{1},...,x_{i},...,x_{n}\right]}^{⊤}\in {\mathbb{R}}^{n\times p}$, and the corresponding label is $Y={\left[y_{1},...,y_{i},...,y_{n}\right]}^{⊤}\in {\mathbb{R}}^{n}$. It is worth noting that in this paper, we divided the brain network into 90 regions of interest (ROI), that is, *N* = 90, so each subject contains a vector of dimension 1 × 4005, which reflects the functional connectivity strength pattern between the 90 brain regions of the subject.

### 3.3 Feature selection using LLM-Lasso

After the above steps, we obtain the FC feature matrix *X* for all subjects. However, this matrix contains a large amount of redundant information, and the number of functional connections between brain regions far exceeds the number of samples, presenting a typical high-dimensional small sample problem. To address this issue and identify the most critical FC features for SZ diagnosis, we employ a penalized Lasso feature selection method. Furthermore, we incorporate the penalty factor derived from the LLM into the Lasso penalty term. This approach not only improves the accuracy of feature selection but also effectively reduces the influence of redundant features, thereby effectively screening key features.

#### 3.3.1 The LLM-Lasso

For the input FC feature *X*∈ℝ^*n*×*p*^ and label *Y*∈ℝ^*n*^, the traditional Lasso method achieves feature selection by introducing an ℓ_1_-norm penalty term in the objective function of minimizing the residual sum of squares (Fonti and Belitser, 2017). The objective function of Lasso regression can be expressed as:


$$\begin{array}{l} \hat{\beta }=\underset{\beta }{\min}\{\frac{1}{2}\sum_{i=1}^{n}{\left(y_{i}-\beta_{0}-x_{i}^{⊤}\beta \right)}^{2}\\ \;+\text{λ}\sum_{j=1}^{p}\left|\beta_{j}\right|\} \end{array}$$ (1)

However, the ℓ_1_ penalty term $\lambda \sum_{j=1}^{p}|\beta_{j}|$ in the above equation imposes the same sparsity constraint on all features, implicitly assuming that all features are equally important in the absence of prior information. This assumption may not hold true when processing high-dimensional brain FC data, as it completely ignores the known biological significance of connections between different brain regions in neuroscience and the differences in their potential associations with schizophrenia. This can lead to feature selection results that deviate from existing understanding of pathological mechanisms.

In order to incorporate domain knowledge into the feature selection process, we can enhance the Lasso method by assigning a penalty factor generated by LLM to each coefficient in the ℓ_1_ penalty. The LLM-Lasso objective function we constructed can be expressed as:


$$\begin{array}{c} \hat{\beta }=\underset{\beta }{\min}\{\frac{1}{2}\sum_{i=1}^{n}{\left(y_{i}-\beta_{0}-x_{i}^{⊤}\beta \right)}^{2}\\ \;+\text{λ}\sum_{j=1}^{p}\omega_{j}|\beta_{j}|\} \end{array}$$ (2)

where ω_*j*_>0 is the penalty factor for the *j*-th feature generated based on knowledge of the LLM domain. Our goal is to expand the uniform penalty term $\lambda \sum_{j=1}^{p}|\beta_{j}|$ in the traditional Lasso to a weighted form $\lambda \sum_{j=1}^{p}\omega_{j}|\beta_{j}|$, so that features with stronger neuroscience evidence receive less penalty, thereby giving them priority in feature selection.

#### 3.3.2 LLM penalty factor optimization based on cross-validation

In LLM-Lasso, we use LLM to generate a penalty factor $S=\left[s_{1},s_{2},...,s_{p}\right]\in {\mathbb{R}}^{p}$ for each feature to guide feature selection for Lasso regression. However, the penalty factors generated by LLM can be inaccurate or produce so-called hallucinations, information that is false or unreliable. To ensure that the model's reliance on LLM output is data-driven and reliable, we introduce a cross-validation procedure to select the optimal penalty factor transformation.

We first define a finite family of transformation functions *U* that transforms the original penalty factor *S* generated by LLM into the optimal penalty W^*^ finally used for Lasso:


$$\begin{array}{l} \begin{array}{cc} W^{*}=u^{*}\left(S\right), & u^{*}\in U \end{array} \end{array}$$ (3)

where W^*^∈ℝ^*p*^, u^*^ is the optimal transformation functions selected from *U* via *k*-fold cross-validation. Next, we divide the dataset into *k* subsets. For the *i*-th fold, let the training set be $D_{tra}^{\left(i\right)}$ and the penalty factor be given by the transformed *W* = *u*(*S*), which is used to calculate the optimal coefficient $\beta_{i,u\left(V\right)}^{*}$ of the Lasso:


$$\begin{array}{c} \beta_{i,u(S)}^{*}=\underset{\beta }{\min}\{\frac{1}{2}\sum_{(x,y)\in D_{tra}^{\left(i\right)}}{\left(y_{i}-\beta_{0}-x_{i}^{⊤}\beta \right)}^{2}\\ \;+\text{λ}\sum_{j=1}^{p}u{\left(S\right)}_{j}|\beta_{j}|\} \end{array}$$ (4)

Then, use the validation data $X_{val}^{\left(i\right)}$ and $Y_{val}^{\left(i\right)}$ to evaluate the cross-validation loss function L, and finally select the transformation function that minimizes the total validation loss:


$$\begin{array}{l} u^{*}=\underset{u\in U}{\arg\min}\sum_{i=1}^{k}L\left(X_{val}^{\left(i\right)}\beta_{i,u\left(S\right)}^{*},Y_{val}^{\left(i\right)}\right) \end{array}$$ (5)

We define *U* as a range of transformations with penalties representing varying degrees of dependence on LLM generation. In this paper, inspired by Zhang et al. (2025), we order the penalty factors by feature importance, with more important features receiving smaller penalties and less important features receiving larger penalties. Therefore, we adopt the family of inverse importance transformation functions:


$$U=\{u:u{\left(S\right)}_{j}=s_{j}^{\varphi },\varphi \in \{0,1,...,\varphi_{\max}\}$$ (6)

where *s*_*j*_ is the penalty factor generated by the LLM for the *j*-th feature, and φ is a parameter that controls the degree of dependence of the penalty factor. If φ = 0, then the converted penalty is the same as the original Lasso penalty, while if φ is larger, it increases the dependence on the penalty factor generated by the LLM.

By cross-validating, we ensure that the model does not degrade due to reliance on unreliable penalties generated by the LLM. In the worst case, since *U* contains *u*_0_(*W*) = 1, which sets all penalty factors to 1, equivalent to the original Lasso, the LLM-Lasso will never perform worse than the original Lasso in terms of cross-validation loss.

#### 3.3.3 Empirically calibrated prompting for optimizing LLM

Prompting is crucial for guiding the LLM to understand specific prediction tasks and generate high-quality penalty factors. In this work, we employ an empirically calibrated prompting method, combined with existing LLM domain knowledge on functional connectivity abnormalities in schizophrenia, to guide the LLM in generating penalty factors for FC features. For such a large-scale SZ dataset, we use a zero-shot prompting approach and set the LLM's temperature parameter *T* to 0 in all experiments, applying a greedy decoding strategy to ensure deterministic and reproducible output.

**Figure 2:**
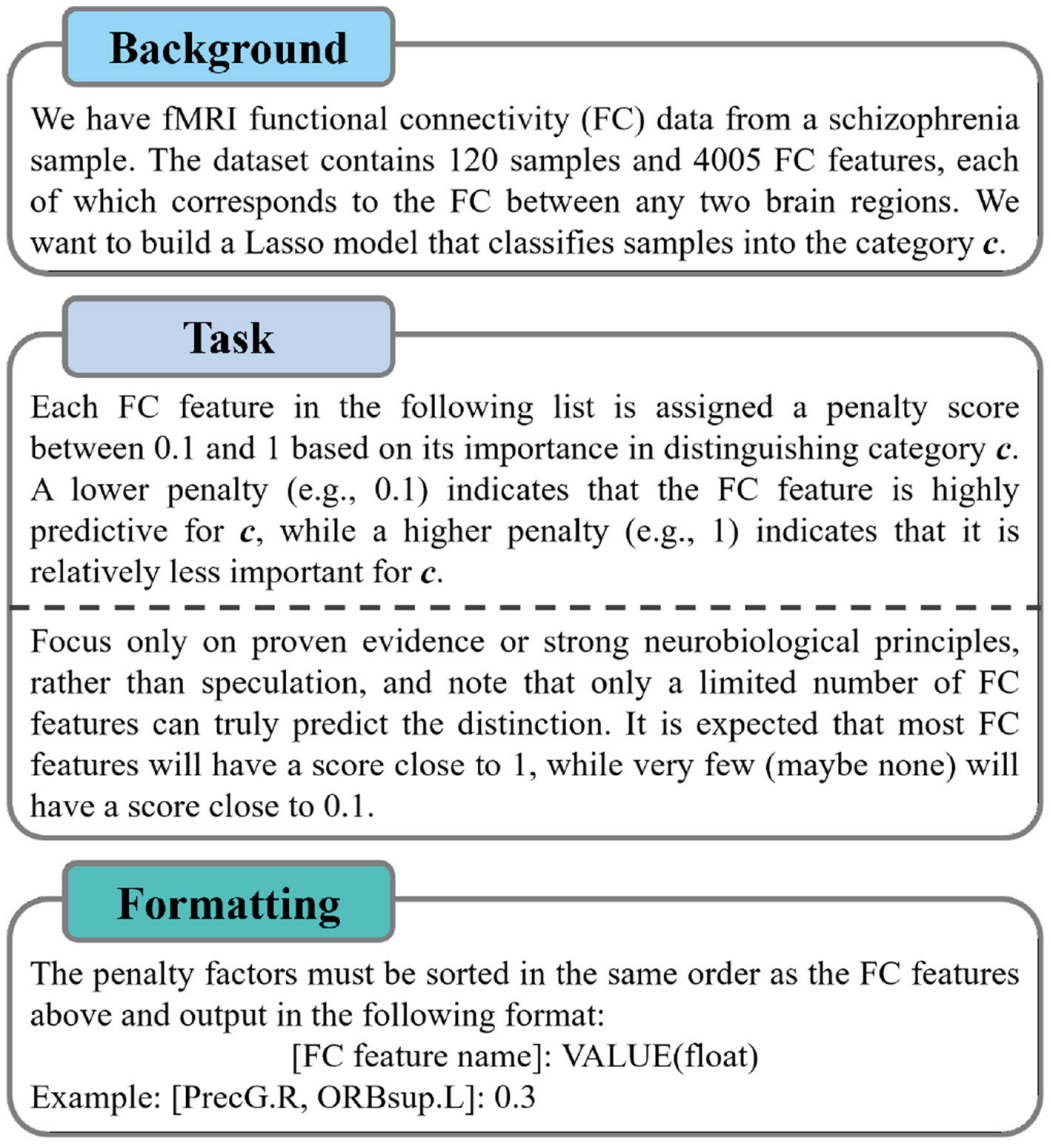
An example is used to describe A.

For all classification tasks, our prompt template consists of *user* and *system*, defined as follows:


$$\begin{array}{l} P=prompt\left(Q^{user}\left(A\left(\phi ,c\right)\right),H^{system}\right) \end{array}$$ (7)

where P is the complete prompt ultimately fed to the LLM, and $Q^{user}$ is the user query. A is the task description, taking as input a feature set ϕ and a category *c*. $H^{system}$ is the system history or dialogue buffer, which maintains the dialogue context. Figure 2 provides an example of the general structure of component A, which typically consists of a background description of the dataset, the assigned task, and formatting instructions.

### 3.4 Explaining counterfactual examples using LLM

To enhance the interpretability of our method, we further introduce a counterfactual explanation model (Mothilal et al., 2020). In this paper, we can formulate a counterfactual statement: *if the abnormal FC between brain regions in SZ patients is adjusted to normal ranges, their condition is closer to that of healthy individuals*. To help patients more intuitively understand these counterfactual examples, we introduce LLM to generate natural language explanations, thereby transforming complex feature changes into easily understandable action recommendations (Fredes and Vitria, 2024).

Before introducing the counterfactual framework, we first represent the FC matrix after feature selection as *X*′∈ℝ^*n*×*q*^, where *q*≪*p*. Furthermore, we train an appropriate machine learning model (i.e., *f*(·)) to predict SZ. In our experiments, we use a support vector machine (SVM) as the classification model due to its strong adaptability to small sample datasets (Xue et al., 2009).

#### 3.4.1 Diversity counterfactual explanation

The input of counterfactual explanation model includes a trained SVM model (i.e., *f*(·)) and the feature vector $m_{i}\in {\mathbb{R}}^{1\times q}$ of the *i*-th subject. Our goal is to generate a set of counterfactual examples $\left\{x_{i}^{1},x_{i}^{2},...,x_{i}^{L}\right\}$ for subject *i* such that its decision outcome $x_{i}^{l}\in {\mathbb{R}}^{1\times q}$ is different from the prediction of the original feature vector *m*_*i*_.

The counterfactual explanation model consists of three parts: loss function *loss*(·), distance function *dist*(·), and diversity metric *diversity*(·). Specifically, the first part pushes counterfactual $x_{i}^{l}$ toward different predictions, the second part makes counterfactual examples closer to the original input, and the third part is used to increase the diversity of counterfactual explanations. In the first part, we use a hinge loss function that helps generate counterfactuals with less variation by reducing the preference for extreme values. The hinge loss is expressed as follows:


$$loss_{hinge}=max(0,1-z\cdot logit(f(x))$$ (8)

where *z* is 1 when Ŷ = 1 and –1 when Ŷ = 0, and *logit*(*f*(*x*)) is the unscaled output of the SVM model. It is worth noting that in our experiments, 1 corresponds to normal subjects and 0 corresponds to patients, so in the verification of converting patients into normal subjects, Ŷ is usually set to 1. *loss*(·) represents the difference between the counterfactual example *x* and the target label Ŷ, and is used to ensure that the generated counterfactual example is consistent with the expected class in the prediction result. For the choice of distance function in the second part, we follow Wachter et al. (2017) proposal and divide the distance of each feature by the median absolute deviation (MAD) of the feature values in the training set:


$$\begin{array}{l} dist\left(x,m\right)=\frac{1}{L}\overset{L}{\underset{\alpha =1}{\sum }}\frac{\left|x^{\alpha }-m^{\alpha }\right|}{MAD_{\alpha }} \end{array}$$ (9)

where *MAD*_α_ is the median absolute deviation of the α-th feature, *L* is the total number of counterfactual examples to generate, *x* represents the counterfactual example and *m* represents the original feature vector. The distance between the counterfactual example *x* and the original example *m* is calculated by *dist*(*x, m*). Its main purpose is to control and prevent the counterfactual example from deviating too much from the original example, thereby ensuring operability. For the third part, we use a determinant-based point procedure to measure the diversity of counterfactual examples, computed by the determinant value of its kernel matrix *K*:


$$\begin{array}{l} diversity=det\left(K\right) \end{array}$$ (10)

where $K_{u,v}=\frac{1}{1+dist\left(x^{u},x^{v}\right)}$, *x*^*v*^ and *x*^*u*^ represent two counterfactual examples. In the experiments, to avoid uncertain determinants, we add small random perturbations on the diagonal elements to calculate the determinant. The purpose of *diversity*(·) is to ensure that the generated counterfactual examples are diverse in the feature space, rather than producing a set of highly similar results. The diversity constraint does not affect the feasibility of individual counterfactual examples, but rather, by controlling the differences between them, it allows us to obtain multiple feasible explanatory paths.

To ensure the validity of the generated counterfactual examples, our optimization process ensures that each counterfactual example meets the following conditions: (i) Valid prediction change (Equation 8): Each counterfactual example's prediction for the target class Ŷ differs from the original input, ensuring the desired classification change is achieved. (ii) Similarity to the original example (Equation 9): Through distance loss *dist*(·), each counterfactual example maintains sufficient similarity to the original example, avoiding excessive feature perturbation and ensuring that the generated counterfactual examples are actionable and consistent with reality. (iii) Diversity (Equation 10): By introducing a diversity metric *diversity*(·), we ensure that the generated counterfactual samples are differentiated in the feature space, providing multiple different interpretation paths, rather than just multiple approximate modifications of the same input. Finally, we can obtain counterfactual examples by optimizing the following loss:


$$\begin{array}{l} \begin{array}{c} X\left(m_{i}\right)=\frac{\gamma_{1}}{L}\overset{L}{\underset{l=1}{\sum }}dist\left(x_{i}^{l},m_{i}\right)\\ -\gamma_{2}diversity\left(x_{i}^{1},x_{i}^{2},...,x_{i}^{L}\right)\\ +\underset{x_{i}^{1},x_{i}^{2},...,x_{i}^{L}}{\arg\min}\frac{1}{L}\overset{L}{\underset{l=1}{\sum }}loss_{hinge}\left(f\left(x_{i}^{l}\right),Ŷ\right) \end{array} \end{array}$$ (11)

where *X*(*m*_*i*_) is the optimization objective function for counterfactual examples, γ_1_ and γ_2_ are hyperparameters for balancing the three parts of the loss function. The above formula reveals the minimum change required for the input data to achieve the idealized result. By adjusting the FC values between abnormal brain regions of SZ patients, their state may be closer to normal.

#### 3.4.2 Using LLM to explain counterfactuals in natural language

**Figure 3:**
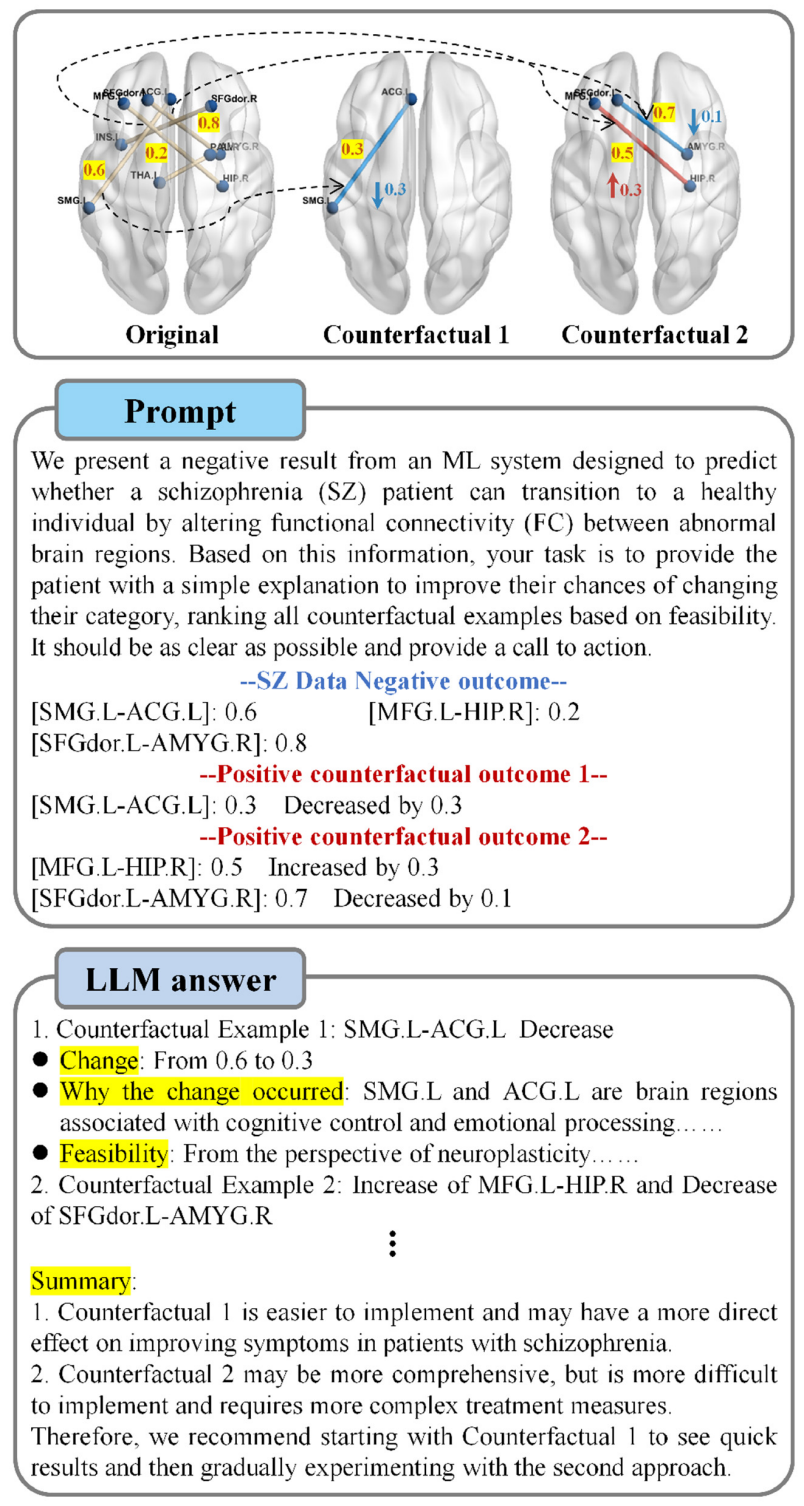
Examples of using LLM to explain counterfactual.

After generating counterfactual examples, our goal is to infer the primary causes from them. To achieve this, we provide the LLM with a set of counterfactual examples (i.e., the FC features to be adjusted) along with the original FC features. We ask it to generate a final explanation in simplest terms, highlighting the steps the patient can take to transition to the healthy category. Specifically, when multiple steps are presented, the LLM ranks these steps based on their potential effectiveness and feasibility, providing the optimal solution. Figure 3 shows an example of the prompts used and the output generated by the LLM. This intuitive explanation approach can provide patients and doctors with the guidance they need to treat the disease.

## 4 Experiments and results

### 4.1 Experimental setting

In this work, we use a support vector machine (SVM) classifier to perform the classification task on five SZ datasets. During the experiments, we evaluate the performance of different methods based on diagnostic accuracy (ACC = $\frac{\text{TP+TN}}{\text{TP+TN+FP+FN}}$), sensitivity (SEN = $\frac{\text{TP}}{\text{TP+FN}}$) and specificity (SPE = $\frac{\text{TN}}{\text{TN+FP}}$). FP, TP, FN, and TN represent false positives, true positives, false negatives, and true negatives, respectively. To ensure fairness, all compared feature selection methods use SVM classifiers. The LLMs we use include GPT-5, GPT-4.1 (Achiam et al., 2023), DeepSeek-V3.2 (Liu et al., 2025), Kimi-k2 (Team et al., 2025), and Gemini 2.5 Pro (Comanici et al., 2025), all of which are accessible through the OpenAI API. Given GPT-5's superior performance, we use the GPT-5 model in subsequent experiments. For an ablation study of the performance of these LLMs, please refer to Section 4.7. For each sample, we set the number of counterfactual examples *L* to be generated to 5, and the hyperparameters γ_1_ = 0.5 and γ_2_ = 1. Notably, we use a five-fold cross-validation strategy in all experiments and in the selection of hyperparameters.

### 4.2 Statistical analysis of FC features

**Figure 4:**
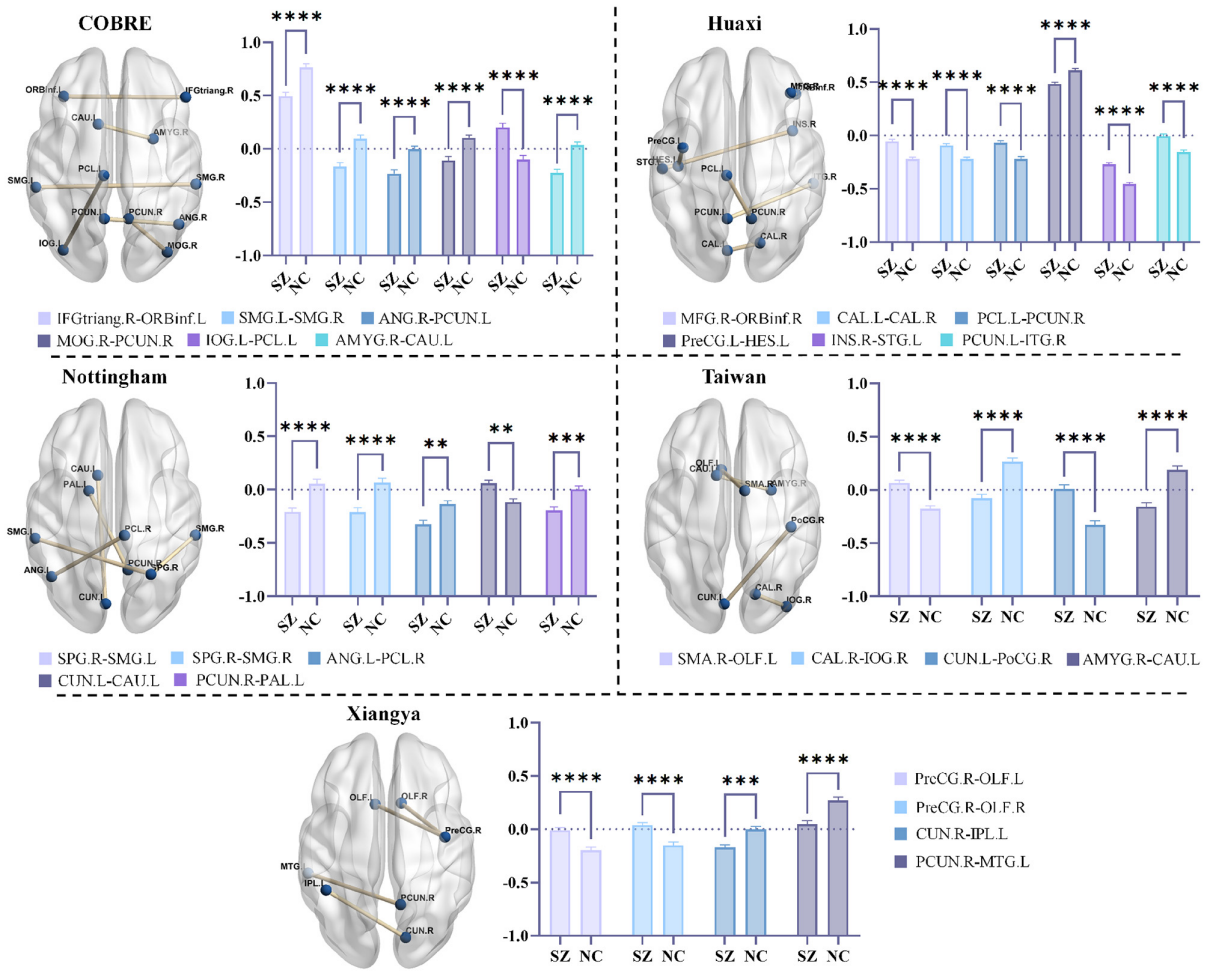
Functional connectivity (FC) retained after feature selection by our method in five datasets and statistical analysis. Among them, ^*^ indicates 0.01 < *p* < 0.05, ^**^ indicates 0.001 < *p* < 0.01, ^***^ indicates 0.0001 < *p* < 0.001, and ^****^ indicates *p* < 0.0001.

In this set of experiments, we perform statistical analysis on the FC remaining after LLM-Lasso feature selection to demonstrate the effectiveness of our method. For intuitiveness, we show in Figure 4 the FC features retained by our method after feature selection on five datasets. As shown in Figure 4, through comprehensive analysis of the five datasets, we find significant FC abnormalities between SZ patients and NC, particularly in key brain regions such as the default mode network (DMN), sensorimotor network, and limbic system. FC abnormalities in these brain regions are closely associated with multiple core symptoms of SZ. For example, DMN regions (such as the PCUN and ANG) are associated with functions such as self-referential processing and mind wandering, and their abnormal connectivity is believed to underlie the neural basis of self-disorder and cognitive decline in SZ (Whitfield-Gabrieli et al., 2009; Tang et al., 2025). Abnormal connectivity between the INS and STG may reflect dysfunction in the salience network. As a core node in this network, dysfunction in the INS may lead to patients' misattribution of external stimuli, thereby triggering symptoms such as hallucinations and delusions (Palaniyappan and Liddle, 2012). Furthermore, abnormal FC between the AMYG and CAU has been detected in multiple datasets and is highly correlated with negative symptoms and motivational deficits in SZ (Arnedo et al., 2015). Abnormal connectivity between the PreCG and OLF suggests sensorimotor integration disorders, consistent with early anosmia and motor planning deficits in SZ (Li et al., 2018). Overall, these results demonstrate that our method can effectively extract stable and biologically meaningful FC features, helping to improve the accuracy and interpretability of SZ classification.

### 4.3 Comparison methods

We compare our proposed method with eight feature selection methods, including two state-of-the-art LLM-based methods (LLM-Select and LLM4FS), four traditional data-driven methods (Lasso, HSIC-Lasso, XGBoost, and Random feature selection), and two deep learning methods (CCNN and DeepFS), to comprehensively evaluate its performance in feature selection tasks.

The details are as follows: (i) LLM-Select (Jeong et al., 2024): A pure text-driven feature selection method that prompts the LLM to output feature importance scores. (ii) LLM4FS (Li and Xiu, 2025): A hybrid feature selection method that allows the LLM to directly call traditional algorithms to analyze sample data and output feature scores. (iii) Lasso (Tibshirani, 1996): A standard Lasso regression model without the LLM. (iv) HSIC-Lasso (Yamada et al., 2014): A kernel-based nonlinear feature selection method that uses the Hilbert-Schmidt independence criterion (HSIC) to measure the relevance of features to the target. (v) Xgboost (Chen and Guestrin, 2016): An embedded feature selection method that selects high-contribution features by training an XGBoost model and extracting feature importances. (vi) Random feature selection (RFS): A baseline method that randomly extracts a subset of features. (vii) CCNN (Meszlényi et al., 2017): Connectome Convolutional Neural Network, used for feature selection in FC. (viii) DeepFS (Li et al., 2023): Deep Feature Screening, uses deep learning to extract low-dimensional representations and perform feature selection.

For all of the above methods, hyperparameters and the LLM (LLM-Select: GPT-4.1, LLM4FS: GPT-4.5) used are set according to the values recommended in the original papers.

### 4.4 Classification performance

In this set of experiments, we compare our proposed method with eight methods and show the results in Table 2. It is not difficult to see that our method shows excellent stability and consistency on all five datasets. Specifically, across the five datasets, our method achieves ACC of 91.67% (COBRE), 85.48% (Huaxi), 90.48% (Nottingham), 88.46% (Taiwan), and 86.21% (Xiangya), respectively. Most methods achieve ACC below 85%. Furthermore, our method demonstrates outstanding performance in both SEN and SPE, achieving 90.91% SPE on the COBRE dataset and 92.00% SEN on the Huaxi dataset. This demonstrates the robustness of our method in distinguishing positive from negative samples. LLM4FS also performs well in SPE, achieving 90.91% and 81.25% on the COBRE and Huaixi datasets, respectively, exceeding other methods and demonstrating its strong ability to discriminate against negative samples. Furthermore, CCNN's performance only outperformed traditional methods, not the two LLM-based methods. However, DeepFS outperformed LLM-Select in terms of ACC on the COBRE, Taiwan, and Xiangya datasets, especially on the Taiwan dataset, where its SPE value of 87.62% surpassed all other methods. We also noted that LLM-based feature selection methods (LLM-Select and LLM4FS) perform well overall, significantly outperforming several other traditional methods. These results indicate that the LLM-based feature selection method not only has strong theoretical significance, but can also provide more reliable support for the diagnosis of complex diseases such as SZ in practical applications.

**Table 2:** Classification performance comparison with existing methods.

**Datasets**	**Metric**	**RFS**	**Lasso**	**Xgboost**	**HSIC-Lasso**	**CCNN**	**LLM-select**	**DeepFS**	**LLM4FS**	**Our method**
COBRE	ACC(%)	62.50	70.83	75.00	79.17	81.12	83.33	84.17	87.50	**91.67**
	SEN(%)	72.73	63.64	77.78	82.35	75.00	86.67	86.11	84.62	**92.31**
	SPE(%)	53.85	76.92	73.33	71.43	86.54	77.78	82.09	**90.91**	**90.91**
Huaxi	ACC(%)	66.13	74.19	75.81	77.42	78.42	80.65	80.58	82.26	**85.48**
	SEN(%)	58.33	75.86	78.12	83.87	76.06	80.56	80.95	83.33	**92.00**
	SPE(%)	76.92	72.73	73.33	70.97	80.88	80.77	80.26	**81.25**	81.08
Nottingham	ACC(%)	64.29	66.67	76.19	78.57	79.25	80.95	80.19	85.71	**90.48**
	SEN(%)	66.67	72.73	84.62	75.00	77.67	85.71	75.47	87.50	**91.67**
	SPE(%)	62.50	60.00	62.50	83.33	80.73	78.57	84.91	83.33	**88.89**
Taiwan	ACC(%)	67.61	69.23	76.92	80.77	80.31	81.48	83.94	84.62	**88.46**
	SEN(%)	69.23	68.75	75.00	84.62	84.04	78.43	79.55	88.89	**90.91**
	SPE(%)	65.62	70.00	77.78	76.92	76.77	84.21	**87.62**	82.35	86.67
Xiangya	ACC(%)	68.97	72.09	75.86	76.74	77.25	81.40	82.01	82.76	**86.21**
	SEN(%)	60.00	66.67	73.33	68.18	80.23	77.78	82.29	81.82	**85.71**
	SPE(%)	78.57	73.53	78.57	85.71	74.76	84.00	81.72	83.33	**86.36**

The best results are in **bold**.

### 4.5 Counterfactual explanation

**Figure 5:**
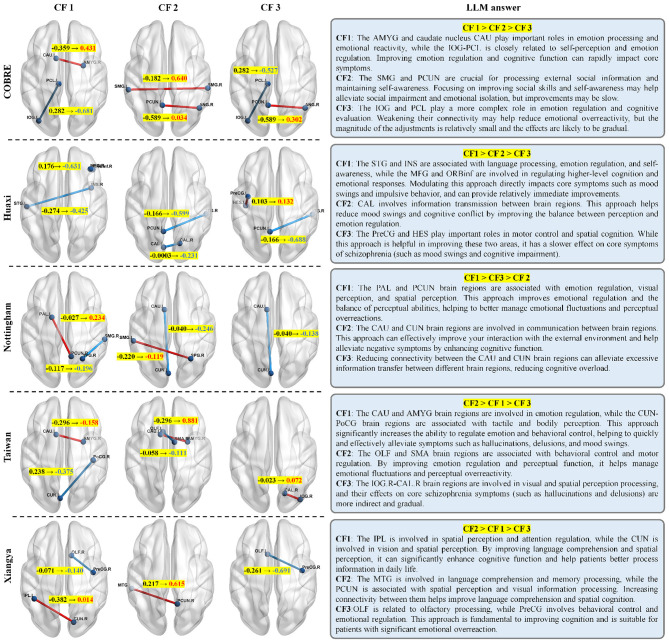
Examples of counterfactuals (CFs) are generated for randomly selected SZ patients from five datasets, along with their final interpretations generated by LLM. Only three counterfactuals are shown here, the remaining FC features after feature selection are in Figure 4. Red indicates increases in FC values between corresponding regions, while blue indicates decreases. The numerical changes between each pair of regions are highlighted. Note that due to space constraints, the final interpretations generated by LLM are abbreviated, with only the key details included.

In this set of experiments, we demonstrate how a counterfactual explanation model generates a set of intuitive and diverse counterfactual (CF) examples for patients. We provide counterfactual explanations by fine-tuning the abnormal FC values of the patients, specifically adjusting the FC values between regions to make the patient's state more similar to that of a healthy individual. As shown in Figure 5, we generate three different sets of CF examples for SZ patients and present them as brain maps. Furthermore, we feed these three sets of CF examples into the LLM, which generates the final intuitive explanations for the patients.

It is evident from Figure 5 that we can bring the patient's condition close to that of a healthy individual by only slightly adjusting the FC values between the corresponding regions. Specifically, in the COBRE dataset, CF1 increases the FC value between CAU.L and AMYG.R from –0.359 to 0.431, and decreases the FC value between PCL.L and IOG.L from 0.282 to –0.681. The changes in CF2 and CF3 are similar to CF1, with no more than two connections adjusted. Similarly, across the five datasets, only two connections are adjusted. For datasets retaining only four features (such as Xiangya and Taiwan), most CF examples only require a single connection change. Furthermore, we find that the magnitude of the FC changes after counterfactual explanation remains stable within 1, indicating that these adjustments have a localized and controllable impact on the patient's brain FC, contributing to a stable state transition. In summary, our method not only helps clinicians identify key FC abnormalities but also provides powerful support for clinical diagnosis.

### 4.6 Cross-dataset validation

To further validate the generalization ability of our proposed method on different datasets, we design this set of experiments. Specifically, we select five datasets: COBRE, Huaxi, Nottingham, Taiwan, and Xiangya. We train on any four of these datasets and test on the remaining one. This experiment is repeated five times, with each dataset serving as the test set in turn, to ensure the robustness and reliability of the evaluation results.

**Figure 6:**
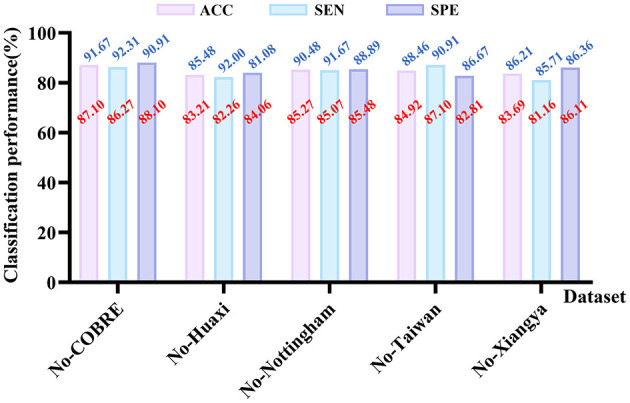
This demonstrates the performance of our method across datasets. **No-** indicates that the dataset is used for testing but not for training, the corresponding value is shown in red. Blue values represent the performance when using the dataset alone, i.e., when only the dataset is used for training and testing, and the values are consistent with the results in Table 2.

The experimental results are shown in Figure 6. As can be seen, our method still exhibits good performance in cross-dataset testing. Although the performance across datasets decreases compared to training and testing on a single dataset only, the overall ACC remains above 83%, demonstrating the model's strong cross-dataset generalization ability. In the No-Xiangya cross-dataset experiment, the SPE for a single dataset is 86.36%, while the SPE for the cross-dataset experiment is 86.11%, showing a very small difference, indicating that our model adapts well to different datasets. Furthermore, the figure also shows other evaluation metrics for cross-dataset testing, with most metrics decreasing by no more than 5%, further validating the robustness and stability of our method. In summary, our proposed method not only achieves good performance on a single dataset but also effectively generalizes across datasets.

### 4.7 Ablation experiments

#### 4.7.1 The impact of different LLMs on the results

**Figure 7:**
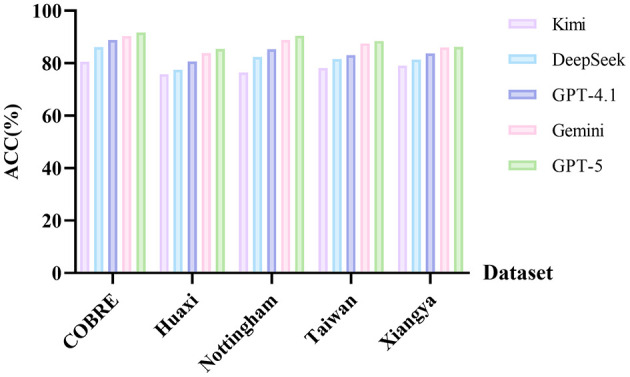
Accuracy of GPT-5, GPT-4.1, DeepSeek-V3.2, Kimi-k2, and Gemini 2.5 Pro on five datasets.

In this set of experiments, we aim to evaluate the impact of the choice of core LLM component on the model's final performance. We compare five mainstream LLMs on the same five datasets: GPT-5, GPT-4.1, DeepSeek-V3.2, Kimi-k2, and Gemini 2.5 Pro. The results are shown in Figure 7.

As shown in Figure 7, the choice of different LLMs significantly impacts overall performance, validating the critical importance of our core component design. Across all five datasets, GPT-5 demonstrates the most stable and superior performance, achieving the highest ACC across all tasks. In particular, on the Huaxi and Nottingham datasets, it achieves improvements of approximately 1.61% and 1.59% over the next-best model, Gemini. DeepSeek performs close to GPT-4.1 on medium-sized datasets (Nottingham and Taiwan), but lags behind by 3.23% on the largest sample, the Huaxi dataset, indicating that its capacity or knowledge density still lags behind the GPT series. Kimi ranks last across all five datasets, with the most significant decline on the Nottingham early SZ task. This is presumably due to the low proportion of psychiatric text in its pre-training corpus, resulting in insufficient prior memory. We believe this phenomenon may be related to the composition of the pre-training data used by each model: GPT and Gemini series extensively incorporate academic and professional web pages (such as arXiv^1^ and PubMed^2^) into their training, while DeepSeek and Kimi still primarily use general web pages, with relatively limited coverage of specialized text. In summary, while all models possess strong language understanding capabilities, GPT-5, with its stronger contextual modeling capabilities and consistency, became the optimal core component choice for this study.

#### 4.7.2 Comparison of LLM-Lasso and Lasso in FC selection

**Figure 8:**
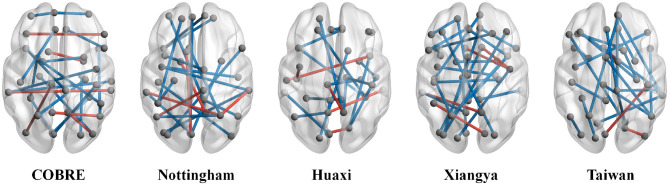
The differences between our proposed LLM-Lasso and the traditional Lasso in the number and overlap of FC features. Red connections indicate that the FC features screened by the two methods overlap, while blue connections indicate the FC features screened by the Lasso method. The FC features screened by LLM-Lasso can be seen in Figure 4.

To evaluate the effectiveness of the LLM-Lasso method and compare it with the traditional Lasso method, we designed an ablation experiment under the same parameter settings, focusing on the differences in the number and overlap of FC features selected by the two methods. The experimental results are shown in Figure 8. We observe that Lasso selects a significantly higher number of FCs than the LLM-Lasso method, indicating that the traditional method tends to select more features, potentially leading to redundant information. Excessive features often result in model overfitting, affecting its generalization ability and diagnostic accuracy. In contrast, the LLM-Lasso method, by incorporating biological knowledge provided by LLM, effectively selected features, focusing on retaining key information related to SZ and avoiding interference from redundant features. Further analysis reveals a certain degree of overlap between the FCs selected by LLM-Lasso and those selected by Lasso, which may be because LLM-Lasso is an improvement upon Lasso. However, it is noteworthy that on the Taiwan dataset, LLM-Lasso identifies a FC (AMYG.R-CAU.L) that traditional Lasso fails to capture. An abnormal FC between AMYG.R-CAU.L has been shown to be highly correlated with SZ-negative symptoms and motivational deficits (Arnedo et al., 2015). This finding suggests that traditional Lasso methods may not adequately consider the biological importance of this connection, while LLM-Lasso, by incorporating domain knowledge, demonstrates greater biological interpretability in feature selection. Overall, LLM-Lasso effectively reduces the influence of redundant information and highlights FCs that are truly clinically significant.

## 5 Discussion

This study proposes a FC analysis method that LLM-based feature selection with counterfactual explanations, demonstrating good feature selection and interpretability on multiple SZ datasets. However, it's important to clarify that this method is currently more suitable as an exploratory brain imaging research tool than a clinically applicable diagnostic test. Although we introduce neuroscience knowledge constraints through LLM, the model still relies on limited samples and heterogeneous scan data, and the counterfactual recommendations have not yet been empirically linked to specific clinical interventions. These factors limit its direct application in personalized diagnosis. In the future, we will validate the stability of this method on larger datasets, gradually promoting its transformation from a research tool to clinical decision support.

Further analysis shows that at the brain network system level, we observe stable consistency across the datasets, with the selected FC features significantly concentrated in key brain regions such as the default mode network, sensorimotor network, and limbic system. However, at the level of specific connectivity edges, different datasets exhibit a certain degree of variability, which may be due to differences in scanning equipment or patient groups across different hospitals. This suggests that while this method can reliably identify which brain network systems may be problematic, it is insufficient to pinpoint the specific FC abnormality in each patient. Future research needs to incorporate more refined clinical stratification and multimodal data to elucidate the biological significance and clinical value of this lateral variability.

In addition, we introduce counterfactual explanations for SZ analysis, but this method has certain limitations. The hyperparameter *L* determines the number of counterfactual examples generated. We chose a fixed value for our experiments, but this choice was not subject to systematic sensitivity analysis or optimization. We recognize that the choice of *L* may affect the results of counterfactual example generation. We hypothesize that a small *L* value may lead to insufficient diversity among counterfactual samples, while a large *L* value may result in excessive diversity among samples, affecting its practical operability. In future research, we plan to explore further improvements to the counterfactual generation method, particularly in how to more effectively select and optimize the hyperparameter *L*. By dynamically adjusting the value of *L*, we expect to generate more diverse and accurate counterfactual examples, thereby improving the model's explanatory power in complex decision-making scenarios.

## 6 Conclusion

This study proposes an innovative framework that combines LLM-guided feature selection with counterfactual explanation, providing a new method for FC analysis in SZ. By incorporating prior knowledge from LLM into the feature selection process, FC features closely related to clinical diagnosis are prioritized, thereby improving the accuracy and interpretability of feature selection. At the same time, counterfactual explanation enables the generation of actionable recommendations that can assist clinical intervention, further enhancing the practicality and understandability of the model. The method achieves positive experimental results on five real-world SZ datasets. In combination with more brain disease data and clinical cases in the future, the method is expected to provide important support for the early diagnosis and personalized treatment of SZ.

## Data Availability

The original contributions presented in the study are included in the article/supplementary material, further inquiries can be directed to the corresponding author.

## Footnotes

1
https://arxiv.org

2
https://pubmed.ncbi.nlm.nih.gov
